# An Individual Patient Data Meta-Analysis on Characteristics, Treatments and Outcomes of Glioblastoma/ Gliosarcoma Patients with Metastases Outside of the Central Nervous System

**DOI:** 10.1371/journal.pone.0121592

**Published:** 2015-04-10

**Authors:** Sophie Pietschmann, André O. von Bueren, Michael J. Kerber, Brigitta G. Baumert, Rolf Dieter Kortmann, Klaus Müller

**Affiliations:** 1 Department of Radiation-Oncology, University Medical Center Leipzig, Leipzig, Saxony, Germany; 2 Department of Pediatrics and Adolescent Medicine, Division of Pediatric Hematology and Oncology, University Medical Center Goettingen, Goettingen, Lower Saxony, Germany; 3 Department of Radiation-Oncology and Clinical Cooperation Unit Neurooncology, MediClin Robert-Janker-Clinic & University of Bonn Medical Center, Bonn, North Rhine-Westphalia, Germany; Martin Luther University, GERMANY

## Abstract

**Purpose:**

To determine the characteristics, treatments and outcomes of patients with glioblastoma multiforme (GBM) or gliosarcoma (GS) and metastases outside of the central nervous system (CNS).

**Methods:**

PubMed and Web of Science searches for peer-reviewed articles pertaining to GBM/ GS patients with metastatic dissemination were conducted using the keywords gliosarcoma, glioblastoma, GBM, metastasis, metastases and metastatic. Additionally, we performed hand search following the references from the selected papers. Cases with metastases to the CNS were excluded and evaluated in a separate study.

**Results:**

109 articles published between 1928 and 2013 were eligible. They reported on 150 patients. We observed a remarkable increase in the number of cases per decade over time. Median overall survival from diagnosis of metastasis (OS_M+_) was 6.0 ± 0.8 months and median overall survival from initial diagnosis (OS_ID_) 13 ± 2.4 months. On univariate analyses, gender, age, the histological subtype, the time interval between initial diagnosis and diagnosis of metastasis and pulmonary involvement did not influence OS_M+_. We did not observe any substantial treatment progress. A comparison of the present cohort with 84 GBM/ GS patients with exclusive CNS dissemination suggests that metastases outside the CNS are related to a slightly more favorable outcome.

**Conclusions:**

The occurrence of extra-CNS metastasis from GBM/ GS is associated with a dismal prognosis, however it seems to compare slightly favorable to CNS dissemination. Crucial treatment progress has not been achieved over recent decades. A central registry should be considered to consecutively gain more information about the ideal therapeutic approach.

## Introduction

Glioblastoma multiforme (GBM) and gliosarcoma (GS) rarely spread beyond the primary tumor site and dissemination outside the central nervous system (CNS) is even more uncommon. Based on older studies [1–4] Picirilli [5] and Lun [6] et al. estimated that the frequency of its occurrence ranges between 0.4% and 2.0%. Accordingly, the current literature on GBM/ GS with extra-CNS metastasis is mainly limited to single case reports or small case series. Although two systematic reviews aiming to summarize the available data were already published in 2008 (n = 128 patients) [5] and 2011 (n = 88 patients) [6] we still have a limited understanding of the disease. The results of these reviews are partly conflicting and thus a range of relevant questions have remained unanswered. For instance, Picirilli et al. identified a considerably larger patient number (+ 40 patients (45%)) than Lun et al. although their search period was shorter. In contrast, Lun et al. performed a much more detailed analysis of the cases. Outcome in terms of overall survival from initial diagnosis differed remarkably in both cohorts. In the meantime a considerable number of new cases of GBM/ GS have been reported and last but not least opinions about the optimal methodological approach to answer certain questions may differ. We now present an individual patient data (IPD) meta-analysis, which is based on a notably larger number of cases (150 patients) to update and complete the existing knowledge.

## Methods

### Aim of the study

The primary objective of this study was to assess clinical characteristics, treatments and outcomes of GBM/ GS patients with extra-CNS metastases. We aimed to include all cases reported in the literature until April 2013. The secondary objective was to evaluate potential prognostic factors for overall survival after the diagnosis of metastasis (OS_M+_) in an explorative manner.

### Search strategy and selection criteria

#### Identification

In a first step we performed PubMed and Web of Science searches with predefined search terms. We did not use any time or language limitations. Key words were: (gliosarcoma OR glioblastoma OR GBM) AND (metastasis OR metastases OR metastatic). In total, the search engines delivered 1695 hits, which were imported in a reference management software (endnote.com X6.0.1). After removal of duplicates, the number of hits was reduced to 1688.

#### Screening

Titles and abstracts were reviewed by two authors (SP and KM). Minimal requirements for further consideration of a case were diagnosis of primary intracranial GBM/ GS and metastatic spread. GBM/ GS whose primary location was spinal were not eligible as they may show different clinical features [7]. Considering these basic inclusion criteria, 1288 publications were excluded after title screening and another 145 after abstract screening. Hand search following the references from the 255 remaining articles revealed 45 new, hitherto unknown publications, which were added to the pool of papers meriting closer investigation (n = 300). However, two publications had to be excluded from our analysis because the full-text articles were not available despite interlibrary loan.

#### Eligibility

**Fig 1:**
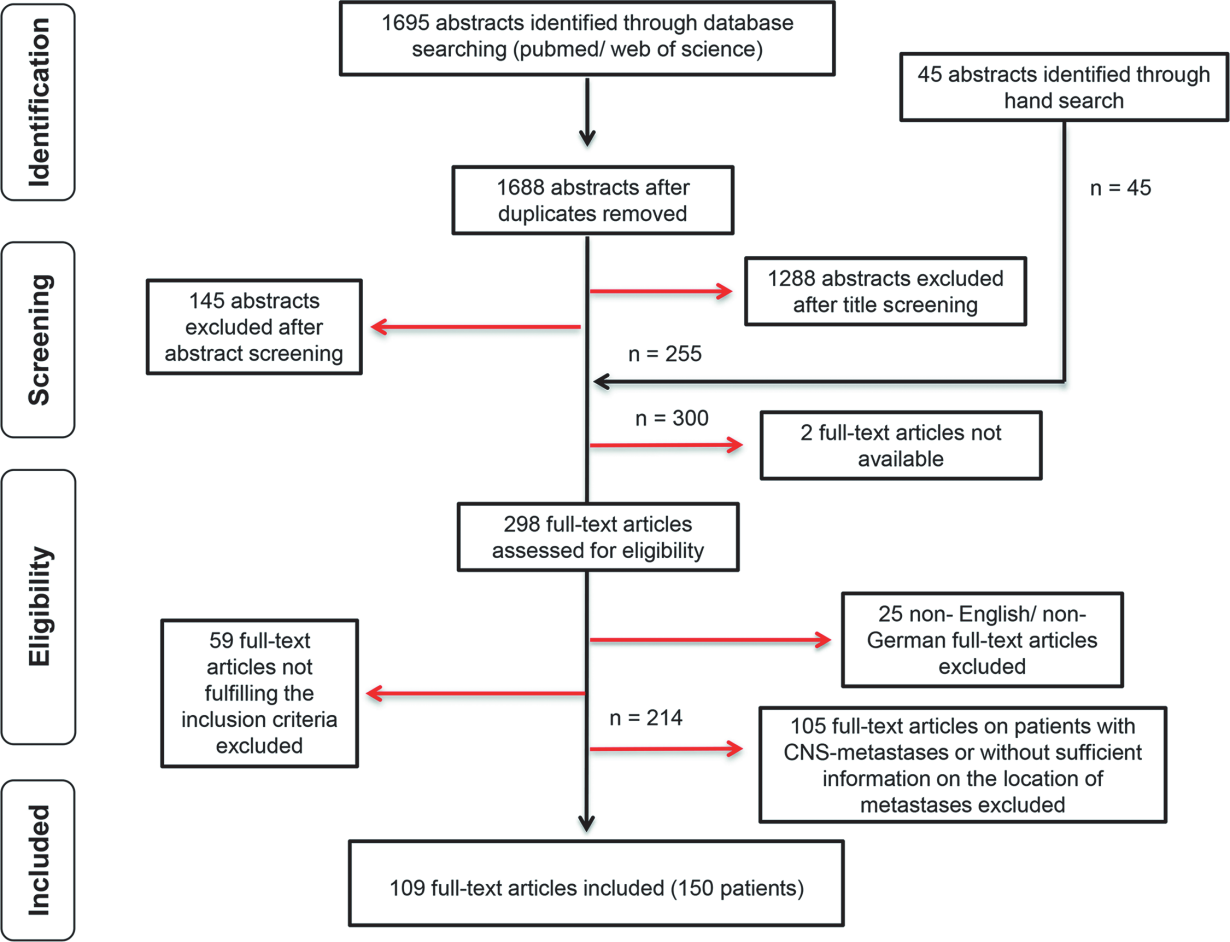
Procedure of publication retrieval and in- and exclusion of cases.

In total, we (SP and KM) evaluated 298 full-text articles for eligibility. Disagreements were resolved through discussion and consensus with a third author (AOvB). Twenty-five articles had to be excluded because they were drafted neither in English nor German and another 59 because they did not fulfill the inclusion criteria. In a second step, we excluded patients with CNS metastases and patients with unclear location of metastases (n = 105 publications). The cases with CNS metastases were analyzed separately and will be reported elsewhere. In total, we included 109 publications. The studies were published between 1928 and 2013 and reported on 150 patients. The procedure of publication retrieval and in- and exclusion of cases is displayed in a PRISMA (Preferred Reporting Items for Systematic Reviews and Meta-Analyses) flow chart (Fig 1) [8].

#### Data extraction

From the eligible articles, the following variables were recorded on a standard data extraction form:

1. survival time divided into three periods:
  - diagnosis of primary tumor to diagnosis of extra-CNS metastasis
  - diagnosis of primary tumor to deaths or to last follow up assessment (OS_ID_)
  - diagnosis of extra-CNS metastasis to deaths or to last follow up assessment (OS_M+_)
2. year of publication
3. age at initial diagnosis
4. gender
5. histology
6. site of metastasis, compiled into
  - thorax and mediastinum (including lungs, pleura and heart)
  - abdomen (including the peritoneal cavity, liver, intestines, spleen, kidney and adrenal gland)
  - bones or bone marrow
  - lymph nodes
  - soft tissue and muscles
  - skin
  - parotid or thyroid gland
  - other locations
7. treatment after diagnosis of metastasis, categorized as
  - not reported or best supportive care
  - surgery only
  - chemotherapy only
  - radiotherapy only
  - radiotherapy + chemotherapy
  - surgery + radiotherapy
  - surgery + chemotherapy
  - surgery + chemotherapy + radiotherapy

### Statistics

Overall survival rates from initial diagnosis (OS_ID_) and diagnosis of metastasis (OS_M+_) were estimated using the Kaplan-Meier method. Survival plots (OS_M+_) relating to categorical variables were compared by means of the log rank test. Additionally, the influence of continuous variables on OS_M+_ was assessed using cox proportional hazards regression analysis. All analyses were conducted using SPSS, version 20.0 (SPSS Inc., Chicago, IL, USA).

## Results

### Frequency of reported cases over time

**Fig 2:**
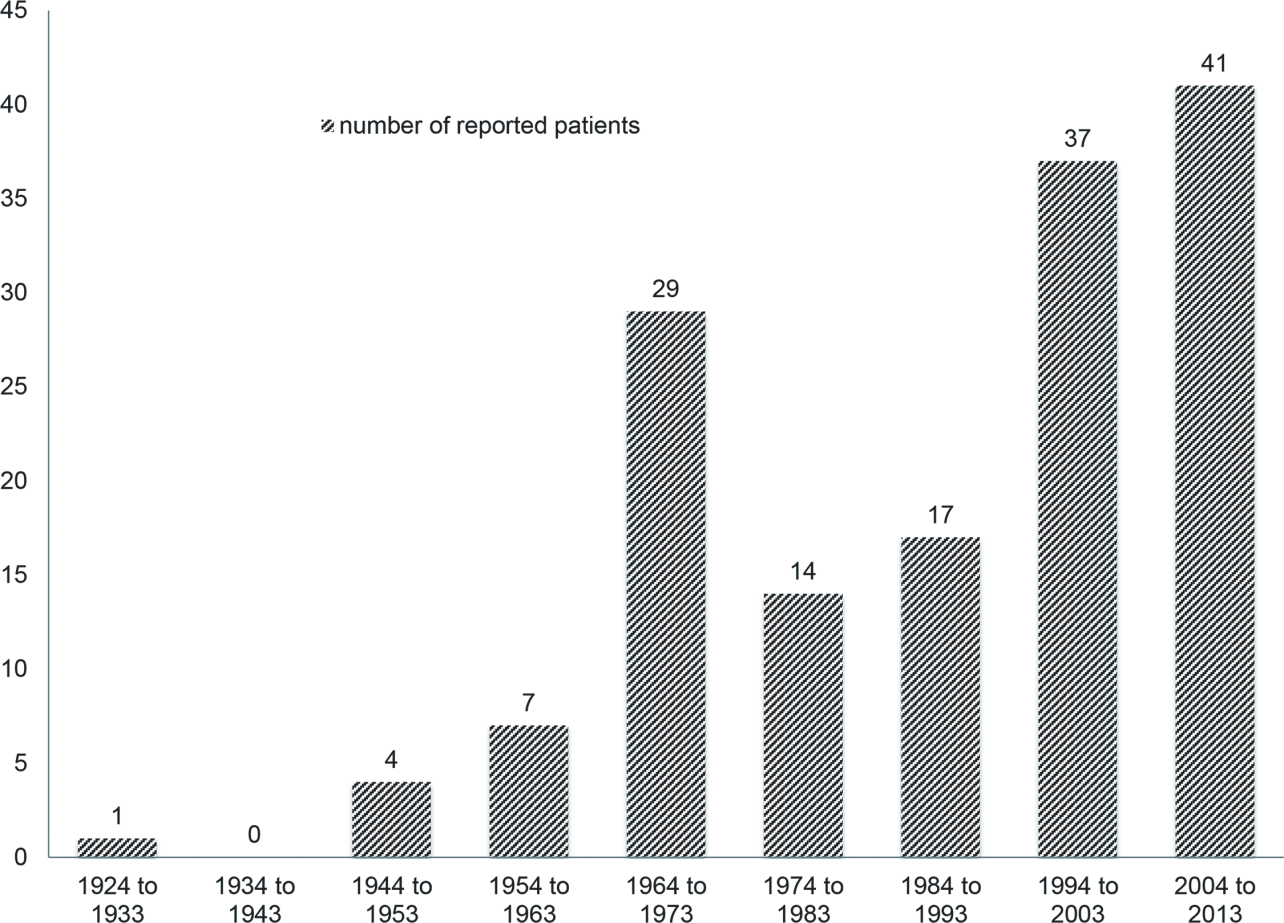
Number of case reports on glioblastomas with extra-CNS metastases over the last decades.

We observed an increase in the number of reported cases over recent decades. More than half of the 150 cases were published after 1993 (Fig 2).

### Clinical characteristics

Gender was unknown in 2/150 cases (1.3%). 105/148 patients (70.9%) were male and 43/148 (29.1%) female. Median age at initial diagnosis was 42 years (range, 4–83 years). Age at initial diagnosis was reported for all patients. Histopathological diagnosis revealed GBM in 137/150 cases (91.3%), GS in 13/150 patients (8.7%). The time interval from initial diagnosis to the occurrence of metastases was reported in 71/150 patients (47.3%). In 7 of these 71 patients (9.9%) primary tumor and metastases were diagnosed simultaneously (Table 1). Median time interval from initial diagnosis to the diagnosis of metastases was 9.0 months (range, 0.0–81.0 months). Metastases affected chest and mediastinum (including lungs, pleura and heart) (52 cases, of those 52 patients 45 had lung metastases), abdomen (including the peritoneal cavity, liver, intestines, spleen, kidney and adrenal gland) (31 cases, of those 31 patients 23 had liver metastases), bones or bone marrow (53 cases), lymph nodes (51 cases), soft tissue and muscles (35 cases), skin (11 cases), parotid or thyroid gland (6 cases) and others (including eye and breast) in 4 cases. Most frequently involved were bones, lymph nodes and lungs. In a considerable number of patients metastasis affected more than one extra-cerebral organ. A wide diversity of different patterns of spread occurred (Table 2).

**Table 1:** Clinical characteristics and treatments after diagnosis of extra-CNS metastasis of 150 GBM/ GS patients reported in literature until April 2013.

	total	%
Male	105/148	70.9
Female	43/148	29.1
Gender not specified	2/150	1.3
Children (≤ 18 years)	11/150	7.3
Adults (> 18 years)	139/150	92.7
GBM	137/150	91.3
GS	13/150	8.7
Primary metastases	7/71	9.9
Secondary metastases	64/71	90.1
Time of dissemination not specified	79/150	52.7
Surgery only	17/60	28.3
Radiotherapy only	4/60	6.7
Chemotherapy only	8/60	13.3
Combination of different treatments	31/60	51.7
Surgery + chemotherapy	2/31	6.5
Surgery + radiotherapy	4/31	12.9
Chemotherapy + radiotherapy	15/31	48.4
Surgery + chemotherapy + radiotherapy	10/31	32.3
Best supportive care only or treatment not specified	90/150	60.0
Death	119/150	79.3

GBM: Glioblastoma multiforme (including one glioblastoma multiforme with an oligodendroglial component), GS: Gliosarcoma.

**Table 2:** Patterns of extracerebral metastasis of 150 GBM/ GS patients reported in literature until April 2013.

Total	%	Patterns of extracerebral metastasis			
		Bone	Lymph node	Lung	Other site
25/150	16.7	•			
20/150	13.3		•		
32/150	21.3				•
13/150	8.7			•	
12/150	8.0	•			•
12/150	8.0		•		•
11/150	7.3			•	•
6/150	4.0	•	•	•	•
5/150	3.3		•	•	
4/150	2.7		•	•	•
3/150	2.0	•	•		
3/150	2.0	•		•	•
2/150	1.3	•		•	
2/150	1.3	•	•		•
0/150	0.0	•	•	•	

#### Treatments after diagnosis of extra-CNS metastasis

The treatment after the diagnosis of extra-CNS metastasis varied widely. Complete treatment details were provided for 60/150 cases (40%). In 90/150 cases (60%) treatment details were not reported or patients exclusively received best supportive care. Seventeen patients were treated with surgery only, four with radiotherapy only, and eight with chemotherapy only. Thirty-one patients underwent a combination of different treatments (surgery + chemotherapy, n = 2; surgery + radiotherapy, n = 4; chemotherapy + radiotherapy, n = 15; surgery + chemotherapy + radiotherapy, n = 10). In total, 33 patients underwent surgery, 33 chemo- and 33 radiotherapy (Table 1).

### Overall survival from initial diagnosis and from diagnosis of extra-CNS metastasis

**Fig 3:**
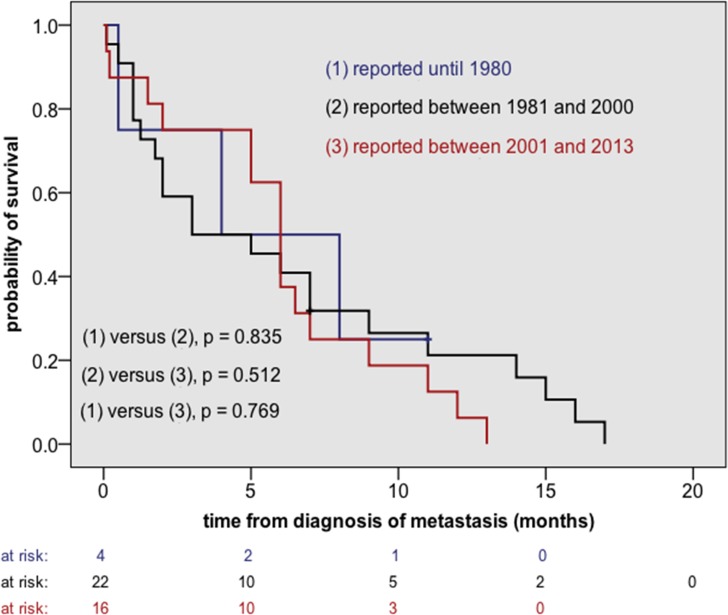
Overall survival after the diagnosis of metastasis (OS_M+_) according to the period of publication. A substantial treatment progress has not been achieved over recent decades.

**Fig 4:**
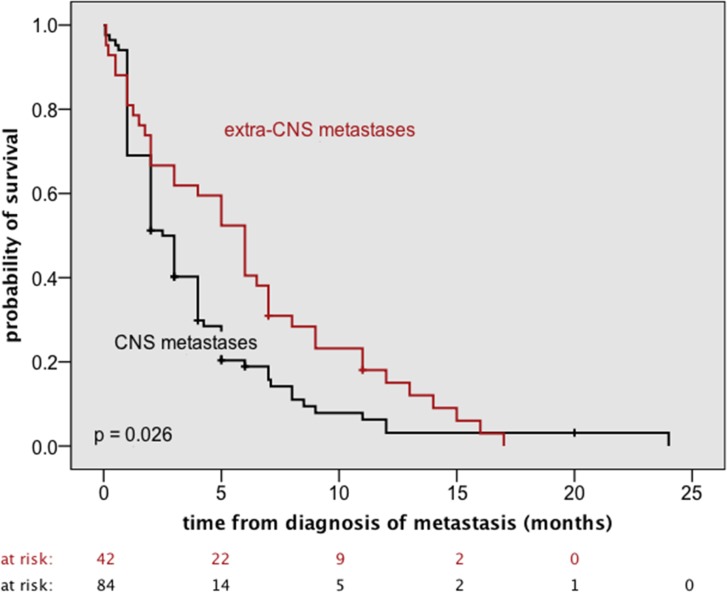
Comparison of OS_M+_ of the present cohort (42 patients) with OS_M+_ of a cohort of GBM/ GS patients with exclusive CNS dissemination (84 patients). Detailed information on characteristics and treatments of these 84 patients and on data collection has been reported previously [9].

110/150 cases provided sufficient information to estimate overall survival from initial diagnosis (OS_ID_) whereas 42/150 cases supplied the relevant data to calculate overall survival from diagnosis of metastasis (OS_M+_). The latter group was used for any further analysis (Table 3 as well as Figs 3 and 4). Median OS_ID_ was 13 ± 2.4 months and median OS_M+_ 6.0 ± 0.8 months.

**Table 3:** Evaluation of potential risk factors for overall survival after diagnosis of extra-CNS metastases (OS_M+_) using Kaplan Meier method and log rank test.

subgroup	n	Deaths	Median OS_M+_ (months)	SE	95% CI	p
male	30	28	6.0	0.9	4.2–7.8	0.935
female	12	12	5.0	2.6	0.0–10.1	
≤ 42 years at ID	22	21	5.0	0.8	3.5–6.5	0.686
> 42 years at ID	20	19	6.0	1.7	2.7–9.3	
< 18 years at ID	4	4	1.3	2.0	0.0–5.1	0.048
≥ 18 years at ID	38	36	6.0	0.8	4.5–7.5	
GBM	37	35	6.0	0.7	4.5–7.5	0.264
GS	5	5	1.5	0.5	0.4–2.6	
time between ID and DoM ≤ 9 months	16	15	4.0	1.3	1.4–6.6	0.866
time between ID and DoM > 9 months	15	15	7.0	1.3	4.5–9.5	
pulmonary involvement at DoM	5	5	1.5	1.5	0.0–4.5	0.156
no pulmonary involvement at DoM	37	35	6.0	0.7	4.5–7.5	

n: number of patients, OS_M+_: overall survival after the diagnosis of metastasis, SE: standard error (months), p: p-value, log-rank test, CI: confidence interval (months), ID: initial diagnosis, GBM: glioblastoma multiforme, GS: gliosarcoma, DoM: diagnosis of metastasis.

### Potential prognostic factors for OS_M+_

On univariate analysis using Kaplan Meier method and log rank test OS_M+_ was not influenced by gender, age at initial diagnosis (cut-off 42 years), histology (GBM versus GS), the time interval between initial diagnosis and metastatic spread (cut-off 9 months) and pulmonary involvement at diagnosis of metastatic disease (Table 3). In contrast, adults showed a more favorable outcome than children, however, the number of patients aged < 18 years was very limited (n = 4) (Table 3). Moreover, we did not find a substantial difference in OS_M+_ between patients reported before 1981, between 1981 and 2000 and after 2000 (Fig 3).

On univariate cox regression analyses age at initial diagnosis (p = 0.852, hazard ratio = 1.002 per year, 95% CI: 0.981–1.024) and the time period between initial diagnosis and diagnosis of metastasis (p = 0.972, hazard ratio = 1.000 per months, 95% CI: 0.980–1.020) did not impact on OS_M+_.

Finally we compared OS_M+_ of the present cohort with OS_M+_ of a cohort of GBM/ GS patients with exclusive CNS dissemination. Detailed information on characteristics and treatments of these patients and on data collection has been reported previously [9]. Median OSM+ was 2.5 ± 0.4 months for the patients with CNS metastases and 6.0 ± 0.8 months for the patients without (Fig 4).

## Discussion

### Is extra-cerebrospinal metastasis from GBM/ GS an increasing phenomenon?

Although extra-cerebrospinal metastasis from GBM/ GS is without doubt a rare phenomenon, the frequency of case reports on the topic has steadily increased over past decades (Fig 2). The reasons for this may be complex including both a rising awareness of the issue in the medical community associated with adaption and extension of staging and significant advances in imaging diagnostics. Moreover, improved local tumor control and prolonged survival, the benefits of combined treatment [10], may have increased the probability of metastatic spread in- and outside the CNS and of occult metastases becoming symptomatic [11, 12].

### Which GBM/ GS patients are at risk for extra-cerebrospinal metastasis?

Interestingly, our data suggests that younger GBM/ GS patients possibly have a predisposition for extra-cerebrospinal metastasis. This observation might be explicable by the fact that younger patients do survive longer [13–16]. Median age in our cohort was 42 years. In the cohort of Lun et al. (n = 88 patients) it was only 38 years [6]. In contrast, median age in a large historical control with non-metastatic glioblastoma patients was 56 years (Stupp et al., 2005, n = 573 patients) [10].

### Has any therapeutic progress been achieved over the last decades?

Moreover, we were interested in whether any advances had been achieved in the treatment of GBM/ GS with extra-cerebrospinal metastases over the last few decades. To answer this question, patients were divided into three groups according to the years of publication of the corresponding articles (≤ 1980, 1981–2000, ≥ 2001). Using the Kaplan-Meier method and the log rank test, we did not detect any substantial difference in OS_M+_ between the three groups (Fig 3), indicating that notable treatment advances had not been achieved over recent decades. Of note, our findings are in contradiction with the results of Lun et al. who reported a progressive lengthening of the interval from detection of extra-cranial metastasis to death from 1940 to 2009, at a rate of 0.7 months per decade. However, Lun et al. evaluated less patients (n = 88) and they used a different methodological approach (linear regression). To use linear regression the authors had to simplify their dataset. Survival intervals were used without identifying the censorship status, which was justified by the small number of censored observations. Interestingly, Lun et al. reported a median OS_M+_ of only 1.5 months for the total of their patients [6]. Median OS_M+_ in our cohort was notably longer (6.0 ± 0.8 months).

### Evaluation of potential non-treatment related prognostic factors for OS_M+_

We assessed the potential prognostic relevance of some simple clinical parameters. Similar to Lun et al. [6], we failed to demonstrate an influence of gender. Age is consistently a strong prognostic factor for non-metastatic glioblastoma [13–16]. Moreover, it has been widely recognized, that there are crucial molecular and clinical differences between adult and pediatric glioblastomas [17]. Hence, we furthermore assessed the impact of age on OS_M+_. We used the median age in our cohort (42 years) and the threshold to adulthood (18 years) as cut-offs for the Kaplan Meier method and log rank test. There was no significant difference in OS_M+_ between patients over 42 years and patients under 42 years. In contrast, children performed poorer than adults. However, the number of children in our analysis was extremely small (n = 4). On univariate cox proportional hazards regression analysis age (as a continuous variable) did not impact OS_M+_. In summary, our findings do not suggest that age is a major prognostic factor for OS_M+_ and support the conclusions previously drawn by Lun et al. [6]. In an older analysis patients with GS and GBM had essentially identical outcomes [18]. Our findings suggest that the assumption, that both entities share a similar prognosis, remains applicable when focusing on the setting of extra-cerebrospinal dissemination. Piccirilli et al. stated that patients with extra-CNS metastasis from GBM with a sarcomatous component had a worse prognosis than patients with other gliomas [5]. In the present dataset we identified only two patients with this particular histological GBM subtype. One of them died two months after diagnosis of metastasis. OS_M+_ of the other patient was not reported [19, 20]. Therefore and given the comprehensive nature of our literature search, we doubt whether the above mentioned statement can be accepted. To the best of our knowledge a potential correlation between the time interval from initial diagnosis of the primary tumor to the diagnosis of extra-cerebrospinal metastases and prognosis has never been assessed before. In our cohort, this time period did not have an influence on OS_M+_. Lun et al. stated that lung metastasis stood out as having the worst prognosis [6]. In contrast, we were unable to confirm this finding, however, this may be due to the small patient number in our analysis (n = 5 patients with pulmonary involvement at initial diagnosis of metastasis) as for statistical reasons, we did not include patients in which lung metastasis occurred at a later point in time (see also “particular statistical limitations”).

### Is it useful to draw any distinction between CNS and extra-CNS metastasis?

In terms of OS_M+_, the present cohort compared favorable with a cohort of 84 GBM/ GS patients with exclusive CNS dissemination. Characteristics and treatments of these patients were reported in detail in a previous article of our working group [9]. As our data do not suggest that the occurrence of (additional) extra-CNS metastasis remarkably worsens prognosis in comparison with exclusive intra-CNS disease, aggressive treatment instead of best supportive care may still be justified in selected patients.

### Which is the best treatment approach in the setting of extra-CNS metastasis?

The question of whether GBM/ GS patients with extra-CNS metastasis are best cared for with aggressive treatment or best supportive care is important and one needs to balance between treatment efficacy in terms of survival, quality of life and toxicity. Lun et al. observed, that patients treated with surgery + radiation + chemotherapy + cerebrospinal fluid shunting had the longest average survival interval from metastasis to death when compared to patients undergoing less intense treatments [6]. Moreover extra-CNS metastasis often affects younger patients in good general condition. Hence, from an ethical point of view, an aggressive treatment approach may be justified whenever feasible. However, one has to keep in mind that currently a survival benefit cannot be proven statistically on the basis of the data available (for details, see particular statistical limitations).

### Limitations inherent to IPD meta-analyses

There are several limitations inherent to IPD meta-analyses. First, there certainly is a selection bias, because the cases reported in the literature might have been published due to their rare or uncommon presentation and outcomes. Second, not all data regarding the patient tumor and treatment characteristics was available for each individual patient. Occasionally the time course of the disease could not be reconstructed. This is reflected by different patient numbers in patient characteristics and survival analyses.

### Particular statistical limitations

In this study, the potential benefit of radio- or chemotherapy and other treatment-related factors for survival was not investigated. The reason for this is that the patients in our cohort received individualized treatments implying that these factors were unknown at the beginning of survival time, i.e. at diagnosis of metastasis. To investigate a variable that is still elusive at the beginning of survival time or that changes over time, time-dependent Cox regression must be used. For example, if we wish to know whether cancer patients’ cumulative dose of chemotherapy affects the length of time until the tumor progresses, we cannot stipulate the cumulative dose as a known quantity at the outset. Patients who survive longer will generally receive a higher total dose. However, this high cumulative dose is not the cause of longer disease control. To allow for this, the cumulative dose must be included in Cox regression as a time-dependent variable. Time-dependent Cox regression is a procedure that requires particularly detailed information about the starting date of therapy, which is generally not provided by case series/ reports extracted from literature [21].

Moreover, we restrained from using a multivariate Cox proportional regression model to reassess the total of potential risk factors for OS_M+_. First, the number of suitable patients (death and complete information on all risk factors) was too small (n = 30) to include all of them simultaneously in the model and second the assumption of proportional hazards, a necessary prerequisite for Cox regression could not be upheld for all factors after visual comparison of the respective Kaplan-Meier plots [21].

## Conclusions

The increasing number of reported GBM/ GS cases with extra-CNS metastasis over time underscores the need to draw a comprehensive picture. These tumors are associated with a dismal prognosis whereby crucial treatment progress is not evident. A central registry should be considered to consecutively gain more information about the ideal treatment approach.

## Supporting Information

S1 Checklist This analysis was performed according to the PRISMA (Preferred Reporting Items for Systematic reviews and Meta-Analysis) guidelines. (DOC)

S1 Dataset Available data on clinical characteristics, treatment and outcome for each individual patient. No.: number, OS ID: overall survival from initial diagnosis, OS M+: overall survival from diagnosis of metastasis.
(XLSX)

S1 References List of references of the applied Data including DOI or PMID for each article if available. (DOCX)

## References

[1] Bouillot-Eimer S, Loiseau H, Vital A. Subcutaneous tumoral seeding from a glioblastoma following stereotactic biopsy: case report and review of the literature. Clinical neuropathology. 2005 Nov-Dec;24(6):247–51. PubMed PMID: . 16320817

[2] Gamis AS, Egelhoff J, Roloson G, Young J, Woods GM, Newman R, et al Diffuse bony metastases at presentation in a child with glioblastoma multiforme. A case report. Cancer. 1990 Jul 1;66(1):180–4. PubMed PMID: . 2162242 10.1002/1097-0142(19900701)66:1<180::aid-cncr2820660132>3.0.co;2-m

[3] Pasquier B, Pasquier D, N'Golet A, Panh MH, Couderc P. Extraneural metastases of astrocytomas and glioblastomas: clinicopathological study of two cases and review of literature. Cancer. 1980 Jan 1;45(1):112–25. PubMed PMID: . 6985826 10.1002/1097-0142(19800101)45:1<112::aid-cncr2820450121>3.0.co;2-9

[4] Smith DR, Hardman JM, Earle KM. Metastasizing neuroectodermal tumors of the central nervous system. Journal of neurosurgery. 1969 Jul;31(1):50–8. PubMed PMID: . 4307543 10.3171/jns.1969.31.1.0050

[5] Piccirilli M, Brunetto GM, Rocchi G, Giangaspero F, Salvati M. Extra central nervous system metastases from cerebral glioblastoma multiforme in elderly patients. Clinico-pathological remarks on our series of seven cases and critical review of the literature. Tumori. 2008 Jan-Feb;94(1):40–51. PubMed PMID: 18468334. 18468334 10.1177/030089160809400109

[6] Lun M, Lok E, Gautam S, Wu E, Wong ET. The natural history of extracranial metastasis from glioblastoma multiforme. Journal of neuro-oncology. 2011 Nov;105(2):261–73. PubMed PMID: 10.1007/s11060-011-0575-8 21512826

[7] Tendulkar RD, Pai Panandiker AS, Wu S, Kun LE, Broniscer A, Sanford RA, et al Irradiation of pediatric high-grade spinal cord tumors. International journal of radiation oncology, biology, physics. 2010 Dec 1;78(5):1451–6. PubMed PMID: 10.1016/j.ijrobp.2009.09.071 20346593 PMC5095924

[8] Moher D, Liberati A, Tetzlaff J, Altman DG, Group P. Preferred reporting items for systematic reviews and meta-analyses: the PRISMA statement. Journal of clinical epidemiology. 2009 Oct;62(10):1006–12. PubMed PMID: 10.1016/j.jclinepi.2009.06.005 19631508

[9] Pietschmann S, von Bueren AO, Henke G, Kerber MJ, Kortmann RD, Muller K. An individual patient data meta-analysis on characteristics, treatments and outcomes of the glioblastoma/gliosarcoma patients with central nervous system metastases reported in literature until 2013. Journal of neuro-oncology. 2014 Aug 27. PubMed PMID: . 25160993 10.1007/s11060-014-1596-x

[10] Stupp R, Mason WP, van den Bent MJ, Weller M, Fisher B, Taphoorn MJ, et al Radiotherapy plus concomitant and adjuvant temozolomide for glioblastoma. The New England journal of medicine. 2005 Mar 10;352(10):987–96. PubMed PMID: . 15758009 10.1056/NEJMoa043330

[11] Kalokhe G, Grimm SA, Chandler JP, Helenowski I, Rademaker A, Raizer JJ. Metastatic glioblastoma: case presentations and a review of the literature. Journal of neuro-oncology. 2012 Mar;107(1):21–7. PubMed PMID: 10.1007/s11060-011-0731-1 21964740

[12] Shahideh M, Fallah A, Munoz DG, Loch Macdonald R. Systematic review of primary intracranial glioblastoma multiforme with symptomatic spinal metastases, with two illustrative patients. Journal of clinical neuroscience: official journal of the Neurosurgical Society of Australasia. 2012 Aug;19(8):1080–6. PubMed PMID: 10.1016/j.jocn.2011.09.024 22704945

[13] Bertolini F, Zunarelli E, Baraldi C, Valentini A, Del Giovane C, Depenni R, et al Survival in patients with newly diagnosed conventional glioblastoma: a modified prognostic score based on a single-institution series. Tumori. 2012 Nov;98(6):756–61. PubMed PMID: 10.1700/1217.13500 23389363

[14] Fujii O, Soejima T, Kuwatsuka Y, Harada A, Ota Y, Tsujino K, et al Supratentorial glioblastoma treated with radiotherapy: use of the Radiation Therapy Oncology Group recursive partitioning analysis grouping for predicting survival. Japanese journal of clinical oncology. 2010 Aug;40(8):726–31. PubMed PMID: 10.1093/jjco/hyq051 20410057

[15] Lamborn KR, Chang SM, Prados MD. Prognostic factors for survival of patients with glioblastoma: recursive partitioning analysis. Neuro-oncology. 2004 Jul;6(3):227–35. PubMed PMID: . Pubmed Central PMCID: 1871999. 15279715 10.1215/S1152851703000620 PMC1871999

[16] Mirimanoff RO, Gorlia T, Mason W, Van den Bent MJ, Kortmann RD, Fisher B, et al Radiotherapy and temozolomide for newly diagnosed glioblastoma: recursive partitioning analysis of the EORTC 26981/22981-NCIC CE3 phase III randomized trial. Journal of clinical oncology: official journal of the American Society of Clinical Oncology. 2006 Jun 1;24(16):2563–9. PubMed PMID: . 16735709 10.1200/JCO.2005.04.5963

[17] MacDonald TJ, Aguilera D, Kramm CM. Treatment of high-grade glioma in children and adolescents. Neuro-oncology. 2011 Oct;13(10):1049–58. PubMed PMID: Pubmed Central PMCID: 3177659. 10.1093/neuonc/nor092 21784756 PMC3177659

[18] Galanis E, Buckner JC, Dinapoli RP, Scheithauer BW, Jenkins RB, Wang CH, et al Clinical outcome of gliosarcoma compared with glioblastoma multiforme: North Central Cancer Treatment Group results. Journal of neurosurgery. 1998 Sep;89(3):425–30. PubMed PMID: . 9724117 10.3171/jns.1998.89.3.0425

[19] Garret R. Glioblastoma and fibrosarcoma of the brain with extracranial metastases. Cancer. 1958 Sep-Oct;11(5):888–94. PubMed PMID: . 13585341 10.1002/1097-0142(195809/10)11:5<888::aid-cncr2820110504>3.0.co;2-t

[20] Yokoyama H, Ono H, Mori K, Kishikawa M, Kihara M. Extracranial metastasis of glioblastoma with sarcomatous component. Surgical neurology. 1985 Dec;24(6):641–5. PubMed PMID: . 2997942 10.1016/0090-3019(85)90122-3

[21] Zwiener I, Blettner M, Hommel G. Survival analysis: part 15 of a series on evaluation of scientific publications. Deutsches Arzteblatt international. 2011 Mar;108(10):163–9. PubMed PMID: Pubmed Central PMCID: 3071962. 10.3238/arztebl.2010.0163 21475574 PMC3071962

