# Supplementary material for: An Individual Patient Data Meta-Analysis on Characteristics, Treatments and Outcomes of Glioblastoma/ Gliosarcoma Patients with Metastases Outside of the Central Nervous System
Source: PLoS One. 2015 Apr 10;10(4):e0121592. doi: 10.1371/journal.pone.0121592 (PMC4393116; doi:10.1371/journal.pone.0121592)
Supplement: S1 References — (DOCX) [file pone.0121592.s003.docx]

1. Aichholzer M, Mazal PR, Haberler C, et al. Epidural metastasis of a glioblastoma after stereotactic biopsy: case report. Minim Invasive Neurosurg 2001;44:175–7.

DOI: 10.1055/s-2001-18127

1. Allan RS. Scalp metastasis from glioblastoma. J Neurol Neurosurg Psychiatry 2004;75:559.

DOI:10.1136/jnnp.2003.020370

1. al-Rikabi AC, al-Sohaibani MO, Jamjoom A, al-Rayess MM. Metastatic deposits of a high-grade malignant glioma in cervical lymph nodes diagnosed by fine needle aspiration (FNA) cytology--case report and literature review. Cytopathology 1997;8:421–7.

PMID: 9439895

1. ANZIL AP. Glioblastoma multiforme with exgracranial metastases in absence of previous craniotomy - case report. Journal of Neurosurgery 1970;33.

PMID: 4316740

1. Armstrong TS, Prabhu S, Aldape K, et al. A case of soft tissue metastasis from glioblastoma and review of the literature. J Neurooncol 2011;103:167–72.

DOI:10.1007/s11060-010-0370-y

1. Astner ST, Pihusch R, Nieder C, et al. Extensive local and systemic therapy in extraneural metastasized glioblastoma multiforme. Anticancer Res 2006;26:4917–20.

PMID: 17214362

1. Ates LE, Bayindir C, Bilgic B, Karasu A. Glioblastoma with lymph node metastases. Neuropathology 2003;23:146–9.

DOI: 10.1046/j.1440-1789.2003.00490.x

1. Beauchesne P, Soler C, Mosnier JF. Diffuse vertebral body metastasis from a glioblastoma multiforme: a technetium-99m Sestamibi single-photon emission computerized tomography study. J Neurosurg 2000;93:887–90.

DOI:10.3171/jns.2000.93.5.0887

1. Beaumont TL, Kupsky WJ, Barger GR, Sloan AE. Gliosarcoma with multiple extracranial metastases: case report and review of the literature. J Neurooncol 2007;83:39–46.

DOI: 10.1007/s11060-006-9295-x

1. Bekar A, Kahveci R, Tolunay S, Kahraman A, Kuytu T. Metastatic gliosarcoma mass extension to a donor fascia lata graft harvest site by tumor cell contamination. World Neurosurg 2010;73:719–21.

DOI:10.1016/j.wneu.2010.03.015

1. Bouillot-Eimer S, Loiseau H, Vital A. Subcutaneous tumoral seeding from a glioblastoma following stereotactic biopsy: case report and review of the literature. Clin Neuropathol 2005;24:247–51.

PMID: 16320817

1. Brandt M. Problem of brain tumor metastases. Arch Psychiatr Nervenkr Z Gesamte Neurol Psychiatr. 1950;185:594–602.

PMID: 14790762

1. Brust cbKKHHTSKAIY. Widespread extracranial metastases of glioblastoma multiforme. Report of case and clinicopathological review of cases in literature. Bull Tokyo Med Dent Univ 1972.

PMID: 4338365

1. Campora RG, Salaverri CO, Ramirez FV, Villadiego MS, Davidson HG. Metastatic Glioblastoma multiforme in cervical lymph-nodes - report of a case with diagnosis by fine-needle aspiration. Acta Cytologica 1993;37:938–42.

PMID: 8249517

1. Cervio A, Piedimonte F, Salaberry J, et al. Bone metastases from secondary glioblastoma multiforme: a case report. J Neurooncol 2001;52:141–8.

PMID: 11508813

1. Cerame MA, Guthikonda M, Kohli CM. Extraneural metastases in gliosarcoma: a case report and review of the literature. Neurosurgery 1985;17:413–8.

PMID: 4047352

1. Chen L, Xiao H, Xu L, Zou Y, Zhang Y, Xu M. A case study of a patient with gliosarcoma with an extended survival and spinal cord metastases. Cell Biochem Biophys 2012;62:391–5.

DOI: 10.1007/s12013-011-9312-3

1. Chesnut RM, Abitbol JJ, Chamberlain M, Marshall LF. Vertebral collapse with quadraparesis due to metastatic gliobla multiforme: case report and review of the literature. J Neurooncol 1993;16:135–40.

PMID: 8289090

1. Chivukula M, Dincer HE, Biller JA, Krouwer HG, Simon G, Shidham V. FNAB cytology of extra-cranial metastasis of glioblastoma multiforme may resemble a lung primary: A diagnostic pitfall. Cytojournal 2005;2:9.

DOI:10.1186/1742-6413-2-9

1. Chung YH, Wong SL, Huang HY. Endobronchial metastasis of glioblastoma multiforme diagnosed by fiberoptic bronchoscopic biopsy. J Formos Med Assoc 1999;98:133–5.

PMID: 10083770

1. Cross KR CTJ. Intracranial neoplasms with extracranial metastases; report of two cases. J Neuropathol Exp Neurol 1952;11:200–8.

PMID: 14918029

1. Dalmer dBH. Ungewöhnliche Wachstumsform und Metastasierung eines Glioblastoms. Psychiat Neurol med Psychol (Lpz.) 1971;23:167.

PMID: 4330749

1. Datta CK, Weinstein JD, Bland JE, Brager PM, Stewart MA. A case of cervical lymph node metastasis resulting from glioblastoma multiforme. W V Med J 1998;94:276–8.

PMID: 9803886

1. Davis M. Spongioblastoma multiforme of the brain. Ann Surg 1928;87:8–14.

PMID: 17865820

1. Dawar R, Khushalani N, Fabiano A, Qiu JX. A Rare Case of Secondary Gliosarcoma with Extracranial Metastases. Journal of Neuropathology and experimental neurology 2012;71:567–8.

DOI: 10.1016/j.clineuro.2012.06.017

1. Didelot A, Taillandier L, Grignon Y, Vespignani H, Beauchesne P. Concomitant bone marrow metastasis of a glioblastoma multiforme revealed at the diagnosis. Acta Neurochir (Wien) 2006;148:997–1000.

DOI:10.1007/s00701-006-0854-x

1. Dietz R, Burger L, Merkel K, Schimrigk K. Malignant gliomas - glioblastoma multiforme and astrocytoma III-IV with extracranial metastases. Report of two cases. Acta Neurochir Suppl 1981;57:99–105.

PMID: 6267905

1. Dolman CL. Lymph-node metastasis as first manifestation of glioblastoma - case report. Journal of Neurosurgery 1974;41:607–9.

PMID: 4371027

1. El-Gindi S, Salama M, Elhenawy M, Farag S. Metastases of glioblastoma multiforme to cervical lymph-nodes - report of 2 cases. Journal of Neurosurgery 1973;38:631–4.

PMID: 4351224

1. Figueroa P, Lupton JR, Remington T, et al. Cutaneous metastasis from an intracranial glioblastoma multiforme. J Am Acad Dermatol 2002;46:297–300.

PMID: 11807444

1. Forsyth PA, Laing TD, Gibson AW, et al. High levels of gelatinase-B and active gelatinase-A in metastatic glioblastoma. J Neurooncol 1998;36:21–9.

PMID: 9525822

1. Frappaz D, Mornex F, Saint-Pierre G, et al. Bone metastasis of glioblastoma multiforme confirmed by fine needle biopsy. Acta Neurochir (Wien) 1999;141:551–2.

PMID: 10392217

1. Friedman JH, Liu HM, Spremulli E, Calabresi P. Distant metastases from a malignant glioma: unusual complications associated with treatment of a glioblastoma: distant metastases and focal white matter degeneration. J Neurol Neurosurg Psychiatry 1987;50:237–8.

DOI: 10.1136/jnnp.50.2.237

1. GARRET R. Glioblastoma and fibrosarcoma of the brain with extracranial metastases. Cancer 1958;11:888–94.

PMID: 13585341

1. GIOK SIE PEK vdSH. Metastasizing primary cerebral tumor. J Neuropathol Exp Neurol 1959;18:575–9.

PMID: 14446438

1. Gjerdrum LM, Bojsen-Moller M. Case of the month: October 1998 - 61 year old male with brain tumor and oral, lung, and palpebral masses. BRAIN PATHOLOGY 1999;9:421–2.

PMID: 10219754

1. Greif J, Horovitz M, Marmor S. Pleuropulmonary metastasis from an intracranial glioblastoma. Lung Cancer 1998;20:135–7.

DOI: 10.1016/S0169-5002(98)00029-4

1. Gropp A. Über ein metastasierendes “Gliom”. Zeitschrift für Krebsforschung 1955;60:590–6.

PMID: 13300505

1. Guo LM, Qiu YM, Ge JW, Zhou DX. Glioblastoma Multiforme with Subcutaneous Metastases, Case Report and Literature Review. Journal of Korean Neurosurgical Society 2012;52:484–7.

DOI: 10.3340/jkns.2012.52.5.484.

1. Gyepes MT, D'angio GJ. Extracranial metastases from central nervous system tumors in children and adolescents. Radiology 1966;87:55–63.

DOI:10.1148/87.1.55

1. Haddon M, Slavin JD, Spencer RP. Multiple bone metastases in a patient with glioblastoma multiforme. Clin Nucl Med 1989;14:13–4.

PMID: 2540929

1. Houston SC, Crocker IR, Brat DJ, Olson JJ. Extraneural metastatic glioblastoma after interstitial brachytherapy. Int J Radiat Oncol Biol Phys 2000;48:831–6.

DOI: 10.1016/S0360-3016(00)00662-3

1. Hübner F BVRH. Case Reports of Symptomatic Metastases in Four Patients with Primary Intracranial Gliomas. Acta Neurochir (Wien) 2001;143:25–9.

PMID: 11345714

1. Jain N, Mirakhur M, Flynn P, Choudhari KA. Cutaneous metastasis from glioblastoma. Br J Neurosurg 2005;19:65–8.

DOI:10.1080/02688690500081423

1. Jamjoom AB, Jamjoom ZA, NaimUrRahman, AlRikabi AC. Cervical lymph node metastasis from a glioblastoma multiforme in a child: Report of a case and a review of the literature. Annals of Saudi Medicine 1997;17:340–3.

PMID: 17369737

1. Kalokhe G, Grimm SA, Chandler JP, Helenowski I, Rademaker A, Raizer JJ. Metastatic glioblastoma: case presentations and a review of the literature. J Neurooncol 2012;107:21–7.

DOI:10.1007/s11060-011-0731-1

1. Klein O, Marchal J. Intraventricular glioblastoma: a paediatric case report. Br J Neurosurg 2007;21:411–3.

DOI:10.1080/02688690701452776

1. Kleinschmidt-Demasters BK. Diffuse bone marrow metastases from glioblastoma multiforme: the role of dural invasion. Hum Pathol 1996;27:197–201.

PMID: 8617464

1. Kros JM, van den Berge H, Tanghe HL, Bakker SL. Right temporal lobe glioblastoma presenting in the left orbit - Case report. Journal of Neurosurgery 2000;92:702–5.

PMID: 10761663

1. Kühn U, Kohler HH, Jecker P. Rare tumors of the parotid gland. Lymphadenoma of a sebaceous gland and extracranial metastasis from glioblastoma [Seltene Parotistumoren. Talgdrusenlymphadenom und extrakranielle Glioblastommetastase]. HNO 2003;51:417–20.

PMID: 12841168

1. Kumar R, Jain R, Tandon V. Thalamic glioblastoma with cerebrospinal fluid dissemination in the peritoneal cavity. Pediatr Neurosurg 1999;31:242–5.

DOI:10.1159/000028870

1. Lampl Y, Eshel Y, Gilad R, Sarova-Pinchas I. Glioblastoma multiforme with bone metastase and cauda equina syndrome. J Neurooncol 1990;8:167–72.

PMID: 2162916

1. Leifer D, Moore T, Ukena T, Wilner D, Thor A, Hedley-Whyte ET. Multifocal glioblastoma with liver metastases in the absence of surgery. Case report. J Neurosurg 1989;71:772–6.

PMID: 2553882

1. Ley A CDOC. Extracranial metastasis of glioblastoma multiforme. J Neurosurg 1961;18:313–30.

PMID: 13761772

1. Liwnicz BH RLJ. The pathways of extraneural spread in metastasizing gliomas: a report of three cases and critical review of the literature. Hum Pathol 1979;10:453–67.

PMID: 381159

1. Maeda D, Miyazawa T, Toyooka T, Shima K. Temporal gliosarcoma with extraneural metastasis: case report. Neurol Med Chir (Tokyo) 2010;50:343–5.

PMID: 20448433

1. Merkel KH, Lahl F, Dietz R, Burger L. The problem of extracranial metastasis of malignant gliomas. Report of 2 cases and review of the literature [Die Problematik der extrakraniellen Metastasierung maligner Gliome. Bericht von zwei Fallen und Literaturubersicht]. Pathologe 1982;3:127–31.

PMID: 6287449

1. Mesfin FB, Deshaies EM, Patel R, Weaver S, Spurgas P, Popp AJ. Metastatic gliosarcoma with a unique presentation and progression: case report and review of the literature. Clin Neuropathol 2010;29:147–50.

DOI: 10.5414/NPP29147

1. Moon K, Jung S, Lee M, et al. Metastatic glioblastoma in cervical lymph node after repeated craniotomies: report of a case with diagnosis by fine needle aspiration. J Korean Med Sci 2004;19:911–4.

DOI: 10.3346/jkms.2004.19.6.911

1. Moriyama T, Kataoka H, Seguchi K, et al. Establishment and characterization of a new human glioblastoma cell line (MGM-1) with highly motile phenotype. Hum Cell 1997;10:105–10.

PMID: 9234071

1. Mousavi M. Bone marrow metastasis from glioblastoma multiforme. J Med Soc N J 1980;77:904–5.

PMID: 6259365

1. Mujtaba SS, Haroon S, Faridi N. Cervical Metastatic Glioblastoma Multiforme. JCPSP-Journal of the College of Physicians and Surgeons Pakistan 2013;23:160–1.

DOI: 02.2013/JCPSP.160161.

1. Nigogosyan G DPSPJ. Brain tumor with extracranial metastases. Report of two cases. Arch Neurol 1962;6:300–6.

DOI:10.1001/archneur.1962.00450220042007.

1. **Nowotny K KHZJ. Zur Frage der extrakraniellen Metastasierung von Gliomen. Wien Z. Nervenheilk 1951;11:200–8.**
2. O'Conner W, Challa V, Nelson O, Gotlieb S. Extracranial metastases of glioblastoma multiforme confirmed by electron microscopy. Surg Neurol 1977;8:347–9.

PMID: 199955

1. Ogungbo BI, Perry RH, Bozzino J, Mahadeva D. Report of GBM metastasis to the parotid gland. J Neurooncol 2005;74:337–8.

DOI: 10.1007/s11060-005-1480-9

1. Ojeda VJ, Sterrett GF. Cerebral gliosarcoma, pulmonary adenoid-cystic carcinoma, and pulmonary metastatic gliosarcoma: report of an untreated case. Pathology 1984;16:217–21.

PMID: 6087260

1. Pang D, Ashmead JW. Extraneural metastasis of cerebellar glioblastoma multiforme. Neurosurgery 1982;10:252–7.

PMID: 6280098

1. Park CC, Hartmann C, Folkerth R, et al. Systemic metastasis in glioblastoma may represent the emergence of neoplastic subclones. J Neuropathol Exp Neurol 2000;59:1044–50.

PMID: 11138924

1. Pham C, Clarencon F, Ganem G, et al. Spinal cervical metastasis from a glioblastoma multiform treated by percutaneous vertebroplasty: a case report. J Neuroradiol 2011;38:323–5.

DOI: 10.1016/j.neurad.2010.08.006

1. Piccirilli M, Brunetto GMF, Rocchi G, Giangaspero F, Salvati M. Extra central nervous system metastases from cerebral glioblastoma multiforme in elderly patients. Clinico-pathological remarks on our series of seven cases and critical review of the literature. Tumori 2008;94:40–51.

PMID: 18468334

1. Pompili A, Calvosa F, Caroli F, et al. The transdural extension of gliomas. J Neurooncol 1993;15:67–74.

PMID: 8455064

1. Potter CR, Kaufman R, Page RB, Chung C. Glioblastoma multiforme metastatic to the neck. Am J Otolaryngol 1983;4:74–6.

PMID: 6324605

1. Rajagopalan V, El Kamar FG, Thayaparan R, Grossbard ML. Bone marrow metastases from glioblastoma multiforme--A case report and review of the literature. J Neurooncol 2005;72:157–61.

DOI: 10.1007/s11060-004-3346-y

1. Sadik AR, Port R, Garfinkel B, Bravo J. Extracranial metastasis of cerebral glioblastoma multiforme: case report. Neurosurgery 1984;15:549–51.

PMID: 6092991

1. Schejbal V. Metastasizing glioma in childhood. Neoplasma 1962;9:585–92.

PMID: 13991553

1. Schönsteiner SS, Bommer M, Haenle MM, et al. Rare phenomenon: liver metastases from glioblastoma multiforme. J Clin Oncol 2011;29:e668-71.

DOI: 10.1200/JCO.2011.35.9232

1. Senetta R, Trevisan E, Ruda R, Benech F, Soffietti R, Cassoni P. Skin metastases of glioblastoma in the absence of intracranial progression are associated with a shift towards a mesenchymal immunophenotype: report of two cases. Acta Neuropathol 2009;118:313–6.

DOI: 10.1007/s00401-009-0543-y

1. Seo YJ, Cho WH, Kang DW, Cha SH. Extraneural Metastasis of Glioblastoma Multiforme Presenting as an Unusual Neck Mass. Journal of Korean Neurosurgical Society 2012;51:147–50.

DOI: 10.3340/jkns.2012.51.3.147

1. Shuangshoti S, Taecholarn C, Kasantikul V. Metastasizing glioblastoma multiforme diagnosed during life of patients: case report and review of literature. J Med Assoc Thai 1988;71:329–39.

PMID: 2844945

1. Slowik F, Balogh I. Extracranial spreading of glioblastoma multiforme. Zentralbl Neurochir 1980;41:57–68.

PMID: 6258355

1. Smith DR HJEK. Metastasizing neuroectodermal tumors of the central nervous system. J Neurosurg 1969;31:50–8.

PMID: 4307543

1. Snopkowska-Wiaderna D, Zielinski KW, Radek M, Papierz W. Extracerebral metastases of glioblastoma have a different vasculature than primary tumour. A case report of glioblastoma extracranial metastases. FOLIA Neuropathologica 2012;50:413–6.

DOI: 10.5114/fn.2012.32376

1. Solau-Gervais E, Flipo RM, Cotten A, Lecomte-Houcke M, Delcambre B. Metastasis from a glioblastoma and Staphylococcus aureus spondylitis in the same vertebral body. Rev Rhum Engl Ed 1998;65:75–6.

PMID: 9523392

1. **Sperduto CM, Bender GP, Aldape K, Sperduto P. Prolonged Survival and Extracranial Metastases from Glioblastoma: A Case Report, Molecular Genetics and Review of the Literature. American Journal of Clinical Oncology-Cancer Clinical Trials 2008;31:609–10.**
2. Steinbok P, Dolman CL, Goldie JH. Variation in response to CCNU of glioblastoma multiforme in brain and cervical lymph node. Case report. J Neurosurg 1985;62:918–21.

PMID: 2987441

1. Steinberg GK SLCFHJ. Evolution and outcome in malignant astroglial neoplasms of the cerebellum. J Neurosurg 1985;62:9–17.

PMID: 3964859

1. Taha M, Ahmad A, Wharton S, Jellinek D. Extra-cranial metastasis of glioblastoma multiforme presenting as acute parotitis. Br J Neurosurg 2005;19:348–51.

DOI: 10.1080/02688690500305506

1. Templeton A, Hofer S, Topfer M, et al. Extraneural spread of glioblastoma--report of two cases. Onkologie 2008;31:192–4.

DOI:10.1159/000118627

1. Terheggen HG, Muller W. Extracerebrospinal metastases in glioblastoma. Case report and review of the literature. Eur J Pediatr 1977;124:155–64.

PMID: 188658

1. Trattnig S, Schindler E, Ungersbock K, et al. Extra-CNS metastases of glioblastoma: CT and MR studies. J Comput Assist Tomogr 1990;14:294–6.

PMID: 2312862

1. Tuominen H, Lohi J, Maiche A, Tormanen J, Baumann P. Mediastinal metastasis of glioblastoma multiforme evolving from anaplastic astrocytoma. J Neurooncol 2005;75:225–6.

DOI: 10.1007/s11060-005-3395-x

1. Utsuki S, Tanaka S, Oka H, Iwamoto K, Sagiuchi T, Fujii K. Glioblastoma multiforme metastasis to the axis. Case report. J Neurosurg Spine 2005;102:540–2.

PMID: 15796392

1. Vural G, Hagmar B, Walaas L. Extracranial metastasis of glioblastoma multiforme diagnosed by fine-needle aspiration: a report of two cases and a review of the literature. Diagn Cytopathol 1996;15:60–5.

PMID: 8807254

1. Waite KJ, Wharton SB, Old SE, Burnet NG. Systemic metastases of glioblastoma multiforme. Clin Oncol (R Coll Radiol) 1999;11:205–7.

PMID: 10465480

1. Walker DG, Pamphlett R. Prolonged survival and pulmonary metastasis after local cure of glioblastoma multiforme. Journal of Clinical Neuroscience 1999;6:67–8.

DOI: 10.1016/S0967-5868(99)90611-2

1. Wallace CJ, Forsyth PA, Edwards DR. Lymph node metastases from glioblastoma multiforme. AJNR Am J Neuroradiol 1996;17:1929–31.

PMID: 8933881

1. Weaver D, Vandenberg S, Park TS, Jane JA. Selective peripancreatic sarcoma metastases from primary gliosarcoma. Case report. J Neurosurg 1984;61:599–601.

PMID: 6747700

1. Wharton SB, Whittle IR, Collie DA, Bell HS, Ironside JW. Gliosarcoma with areas of primitive neuroepithelial differentiation and extracranial metastasis. Clin Neuropathol 2001;20:212–8.

PMID: 11594506

1. Widjaja A, Mix H, Golkel C, et al. Uncommon metastasis of a glioblastoma multiforme in liver and spleen. Digestion 2000;61:219–22.

DOI:10.1159/000007761

1. WIsiol ES HSFL. Extracranial metastases of a glioblastoma multiforme. J Neurosurg 1962;19:186–94.

PMID: 14007684

1. Yao YT LWHCLK. A case of glioblastoma multiforme with extracranial metastases. J Formos Med Assoc 1975;(74):220–8.

PMID: 167101

1. Yasuhara T, Tamiya T, Meguro T, et al. Glioblastoma with metastasis to the spleen--case report. Neurol Med Chir (Tokyo) 2003;43:452–6.

PMID: 14560851

1. Yilmaz M, Is M, Celikoglu E, Kiraz I, Yavuzer D. Isolated Cutaneous Metastases From an Intracranial Glioblastoma: A Case Report. Neurosurgery Quarterly 2013;23:55–7.

DOI: 10.1097/WNQ.0b013e31825a6ac8

1. Yokoyama H, Ono H, Mori K, Kishikawa M, Kihara M. Extracranial metastasis of glioblastoma with sarcomatous component. Surg Neurol 1985;24:641–5.

PMID: 2997942

1. Yung WK, Tepper SJ, Young DF. Diffuse bone marrow metastasis by glioblastoma: premortem diagnosis by peroxidase-antiperoxidase staining for glial fibrillary acidic protein. Ann Neurol 1983;14:581–5.

PMID: 6316836

1. Zappia JJ, Wolf GT. Cervical metastatic glioblastoma multiforme. Arch Otolaryngol Head Neck Surg 1992;118:755–6.

DOI:10.1001/archotol.1992.01880070085016

1. Zeitlhofer J KH. Extracranial metastases of glioma. Zentralbl Neurochir 1952;12:347–56.

PMID: 13091280

1. Zhen L, Yufeng C, Zhenyu S, Lei X. Multiple extracranial metastases from secondary glioblastoma multiforme: a case report and review of the literature. J Neurooncol 2010;97:451–7.

DOI: 10.1007/s11060-009-0044-9
